# Effect of seasonality on the population density of wetland aquatic insects: A case study of the Hawr Al Azim and Shadegan wetlands, Iran

**DOI:** 10.14202/vetworld.2019.584-592

**Published:** 2019-04-22

**Authors:** Hassan Nasirian, Aref Salehzadeh

**Affiliations:** 1 Department of Medical Entomology and Vector Control, School of Public Health, Tehran University of Medical Sciences, Tehran, Iran; 2 Department of Medical Entomology, School of Medicine, Hamadan University of Medical Sciences, Hamadan, Iran

**Keywords:** change of insect population, effect of seasonality on insect population density, seasonal climate change, wetland aquatic insect

## Abstract

**Aim:**

Wetlands are extremely suitable ecosystems to assess the effect of climate change on the density of aquatic insects. This study aimed to assess the effect of seasonality on populations of aquatic insects in the Hawr Al Azim and Shadegan wetlands.

**Materials and Methods:**

The insect samplings were conducted at a large area of the Hawr Al Azim and five different sites of the Shadegan wetlands. In total, 18,534 arthropods of different life stages, including 12 orders containing 51 families, were collected and identified from the selected sites of the Shadegan and Hawr Al Azim wetlands.

**Results:**

Results showed that the population density of wetland aquatic insects gradually increased as the average daily temperature decreased, positively increased with daily mean relative humidity and precipitation, and decreased with the mean daily evaporation between October and April. Conversely, the population density of wetland aquatic insects gradually decreased with increasing average daily temperature and reduction of the mean relative humidity and precipitation and increasing the average evaporation from April to September. When differences between the average daily and water temperatures reached minimum in April, the population density of wetland aquatic insects reached maximum and turned mainly to families that they have high level of biological indices, indicating that wetlands have clean waters around the spring. While around the autumn conversely, they mostly changed to families that they have low level of biological indices, indicating that wetlands have unclean waters.

**Conclusion:**

The present study showed an optimum condition for the growth of insects around spring. Seasonality affects the population density of wetland aquatic insects during a year.

## Introduction

Seasonality, environment, and climate are regulatory agents for some organisms and short-term (seasonal) trends and long-term patterns may generate cyclic biological fluctuations [1]. They have direct effects on distribution, abundance, and longevity of the vectors; incubation period, replication, and lineage of pathogens; abundance, distribution, and behavior of hosts; and their interactions [2]. In wildlife, another focus of disease ecologists is the effects of seasonality on disease dynamics. Host population dynamics, host physiology, and disease dynamics can be affected by seasonal variation in resource availability, temperature, and precipitation [3-8]. Seasonality and climate affect plants, distribution of vector-borne diseases, and densities of animals [9,10].

Climate change also causes wetland deterioration. In fact, it accelerates plant and animal population and species reduction and promotes biotic homogenization, resulting in an impoverishment of biological abundance and loss of species’ diversity, respectively [11-13]. In the global climate, there is no doubt that the wetlands also will strongly be affected by the predicted changes [14,15]. Wetlands are strongly dependent on the water cycle covering about 6% of the Earth’s surface. They normally located at the interface between aquatic and terrestrial ecosystems [16]. Furthermore, they are known as productive ecosystems, performing significant economic benefits, bearing many socioeconomic advantages, preventing dust phenomena, and reservoirs of animal and insect biodiversity [17-23]. Meanwhile wetlands are extremely vulnerable ecosystems to consider the effects of climate change [16]. It seems they are also suitable ecosystems to assess the effect of climate change on the density of aquatic insects.

The Hawr Al Azim and Shadegan wetlands of Iran are of both global and local importance[24]. In a study conducted by Nasirian *et al*.[21], the organic pollution and water quality of the Hawr Al Azim and Shadegan wetlands were evaluated by biological indices including family biotic index; biological monitoring working party; average score per taxon; *Ephemeroptera*, *Plecoptera*, and *Trichoptera* index; and percent contribution of dominant family using insects. Results showed that the quality of these wetland waters was poor, but the effects of seasonality on the density of aquatic insects and related implications for climate change were not evaluated. The present study was designed to assess the effect of seasonality on populations of aquatic insects in the Hawr Al Azim and Shadegan wetlands.

## Materials and Methods

### Ethical approval

This study was conducted on aquatic insects of the Hawr Al Azim and Shadegan wetlands in Iran and there was no need for permission of ethics committee.

### Wetland details

**Figure-1:**
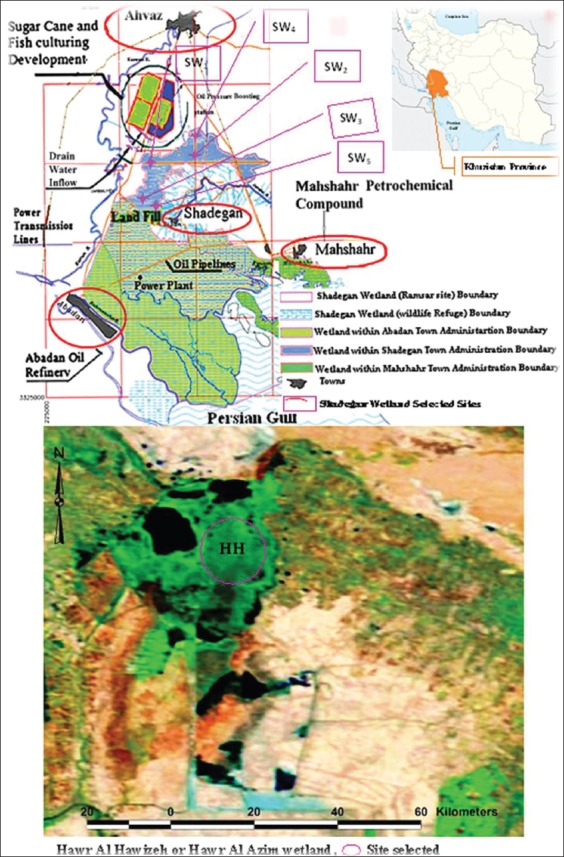
Location map of the Shadegan and Hawr Al Azim wetlands and sites of insect sampling. Source adapted from A: wikipedia.org 2014, B: PCE 2002, and C: persianblog.ir 2014.

This study was performed in the Hawr Al Azim (also known as Hawr Al Hawizea) and Shadegan wetlands in Khuzestan Province, Southwest Iran (Figure-1). In Iran, the Shadegan wetland is the largest wetland, having an area of 537,700 hectares. It is located 52 km from Abadan and 40 km from Ahvaz and is surrounded by Shadegan County and Khor Doraq to the north, Bahmanshir River to the south, Darkhovien and Abadan road to the west, and Khure-Musa to the east. The wetland area coordinates are as follows: 48° 17´-48° 50´E and 30° 17´-30° 58´N. Hawr Al Hawizeh forms one of the largest permanent freshwater wetlands in Lower Mesopotamia. The area is about 56,654 hectares and located in the North Azadegan Plain, 80 km southwest of Ahvaz, near the border between Iran and Iraq. Its area coordinates are as follows: 47° 20´-47° 55´E and 30° 58´-31° 50´N [21,24,25]. Based on analysis of the parameters including salinity, hardness, total dissolved solids, turbidity, electrical conductivity and total suspended solids of water, and biological indices using insects, the water quality of the Shadegan and Hawr Al Azim wetlands relatively had a poor condition [21,26].

### Site selection

The insect samplings were conducted at a large area of the Hawr Al Azim and five different sites of the Shadegan wetlands:

- SW1 – water canal entrance to the Shadegan wetland. The canal receives wastewater output from sugarcane fields and factories.
- SW2 – middle of the Shadegan wetland and relatively remote from the main flow inputs to the wetland.
- SW3 – near Ragbeh and Sarakhieh villages and tourism area on the western side of the Shadegan wetland. Rural and livestock wastewaters are released into the wetland in this area.
- SW4 – north section of Shadegan wetland which receives wastewater from a canal draining sugarcane fields and factories.
- SW5 – near the Shadegan City wastewater outfall [27].

Insect samples were collected from a large area near the middle of the Hawr Al Azim wetland which is relatively unaffected by human activity [21].

### Insect collection

Adult stages of aerial insects were collected using a long-handled wide mesh net. During the same sample dates, a modified student D-form small mesh net was used for collecting adult and premature insect stages in the lotic and lentic zones, as well as from floating aquatic plants. Sampling was done at each site in the months of October and December, 2011, and March, April, June, July, and September, 2012. Adult and premature insect stages were collected by fine forceps after pouring the student D-form net contents into a rectangular plastic container with 15 cm × 30 cm × 45 cm dimensions. All collected specimens were poured into vials containing ethanol 96% and transferred to the entomology laboratory after labeling. At each of the five sites in the Shadegan wetland and within the broad general area of the Hawr Al Azim wetland, samples were collected from ten subsites. All sites in the two wetlands were inundated by water during sample collection, with depths ranging from approximately 0.2 m to 1 m [25]. The specimens were identified under a dissecting microscope using morphology-based identification keys at the entomology laboratory and also were used for calculating biological indices [21].

### Meteorological data

Meteorological data between October 2011 and September 2012 including mean daily temperature, mean relative humidity, and rainfall were obtained from the Shadegan synoptic weather station. Furthermore, mean water temperature was determined by measuring water temperatures at each sample site of the wetlands [28].

### Statistical analysis

Wilcoxon signed-ranks test was applied for comparing monthly abundance of the aquatic insects in the Shadegan and Hawr Al Azim wetlands (Table-1).

**Table-1:** Wilcoxon signed-ranks statistical analyses between monthly abundance of the aquatic insects in the Shadegan and Hawr Al Azim wetlands.

Descriptive					

Month	Mean±standard deviation		Month	Mean±standard deviation	
October	33.1±49.7		June	461.6±463.4	
December	319.3±437.7		July	428.9±655.7	
March	560.7±629.6		September	215.7±330.1	
April	628.4±618.5				

**Wilcoxon signed-ranks**					

**Between months**		**Mean ranks**		**Z**	**p-value (two-tailed)**

		**Negative**	**Positive**		

October	December	0.001	4.0	−2.366^a^	0.018
	March	0.001	4.0	−2.366^a^	0.018
	April	0.001	4.0	−2.366^a^	0.018
	June	0.001	4.0	−2.366^a^	0.018
	July	0.001	4.0	−2.366^a^	0.018
	September	1.0	4.5	−2.197^a^	0.028
December	March	1.0	4.5	−2.197^a^	0.028
	April	2.0	4.8	−1.690^a^	0.091
	June	6.0	3.0	−0.943^a^	0.345
	July	3.3	4.5	−0.676^a^	0.499
	September	3.6	5.0	−0.676^b^	0.499
March	April	4.0	4.0	−0.338^a^	0.735
	June	4.7	3.5	0.0001^c^	1.000
	July	4.0	4.0	−0.338^b^	0.735
	September	5.0	2.7	−1.014^b^	0.310
April	June	4.4	3.5	−0.593^b^	0.553
	July	5.3	3.0	−0.338^b^	0.735
	September	6.0	2.5	−0.676^b^	0.499
June	July	3.4	5.5	−0.507^b^	0.612
	September	4.3	2.0	−2.028^b^	0.043
July	September	4.5	1.0	−2.197^b^	0.028

a Based on negative ranks and

b based on positive ranks,

c The sum of negative ranks equals the sum of positive ranks, p-value of significant (p<0.05) is shown in bold font style

## Results

Tables-2-4 show the monthly abundance, families, and orders or suborders of aquatic insects in the Shadegan and Hawr Al Azim wetland selected sites, respectively. In total, 18,534 arthropods of different life stages including 12 orders containing 51 families were collected and identified from the selected sites of the Shadegan and Hawr Al Azim wetlands (Tables-2-4). From 12 identified orders, nine orders containing 49 families were *Coleoptera*, *Diptera*, *Ephemeroptera*, *Hemiptera*, *Lepidoptera*, *Megaloptera*, *Neuroptera*, *Odonata* (*Anisoptera* and *Zygoptera*), and *Plecoptera*. Three orders also were from the *Malacostraca* (*Crustacea*) class, including *Decapoda* (two families), *Isopoda* (one suborder), and *Mysida* (one genus) (Table-3).

**Table-2:** Abundance (numbers) of aquatic insects in the Shadegan and Hawr Al Azim wetlands, October 2011-September 2012.

Site	2011			2012				Total
	
	Autumn		Winter	Spring		Summer		
			
	October	December	March	April	June	July	September	
SW_1_	66	210	501	617	280	235	132	2041
SW_2_	30	86	581	883	349	330	192	2451
SW_3_	-	259	661	498	405	295	146	2264
SW_4_	81	203	731	474	751	231	155	2626
SW_5_	55	1425	879	1204	402	542	174	4681
HH	-	52	572	723	1044	1369	711	4471
Total	232	2235	3925	4399	3231	3002	1510	18534

SW_1_ to SW_5_=Selected sites of the Shadegan wetland and HH=Hawr Al Azim wetland

**Table-3:** Families of the aquatic insects in the wetlands, October 2011-September 2012.

Order (Suborder)	2011			2012			
	
	Autumn		Winter	Spring		Summer	
			
	October	December	March	April	June	July	September
*Coleoptera*	Curculionidae Dytiscidae Elmidae Haliplidae Hydraenidae Hydrophilidae	Dytiscidae Elmidae Hydraenidae Hydrophilidae	Dytiscidae Elmidae Hydraenidae Hydrophilidae	Chrysomelidae Dytiscidae Gyrinidae Helophoridae Hydraenidae Hydrophilidae	Chrysomelidae Carabidae Curculionidae Dytiscidae Haliplidae Hydraenidae Hydrophilidae Scirtidae (Helodidae) Staphylinidae	Curculionidae Dytiscidae Elmidae Gyrinidae Haliplidae Helophoridae Heteroceridae Hydraenidae Hydrochidae Hydrophilidae	Chrysomelidae Dryopidae Dytiscidae Elmidae Hydraenidae Hydrophilidae Staphylinidae
*Diptera*	Syrphidae	Chironomidae Culicidae Tabanidae	Chironomidae Tabanidae	Chironomidae Dolichopodidae Stratiomyidae	Chironomidae Tabanidae	Chironomidae Tabanidae	Chironomidae Tabanidae
*Ephemeroptera*	Baetidae Caenidae Heptageniidae	Baetidae Caenidae Leptophlebiidae	Baetidae Caenidae Heptageniidae Isonychiidae	Caenidae	Caenidae Leptophlebiidae	Baetidae Caenidae	Baetidae Caenidae
*Hemiptera*	Corixidae Mesoveliidae Notonectidae	Corixidae Gerridae Notonectidae Veliidae	Corixidae	Corixidae Hydrometridae Mesoveliidae Pleidae Saldidae	Belostomatidae Corixidae Gerridae Mesoveliidae Naucoridae Notonectidae Pleidae Veliidae	Corixidae Notonectidae Pleidae	Corixidae Naucoridae Notonectidae Pleidae
*Lepidoptera*	Pyralidae	Pyralidae	—	Pyralidae	Pyralidae	Pyralidae	—
*Megaloptera*	—	—	Corydalidae	—	—	—	—
*Neuroptera*	Sisyridae	—	—	—	—	—	—
*Odonata* (Anisoptera)	Aeshnidae Corduliidae Libellulidae	Aeshnidae Libellulidae	Aeshnidae Libellulidae	Cordulegastridae Libellulidae	Aeshnidae Libellulidae	Aeshnidae Cordulegastridae Corduliidae Libellulidae	Aeshnidae Gomphidae Libellulidae
*Odonata* (Zygoptera)	Coenagrionidae Calopterygidae Lestidae	Coenagrionidae	Coenagrionidae	Coenagrionidae	Coenagrionidae	Coenagrionidae	Coenagrionidae
*Plecoptera*	Perlidae Perlodidae	—	—	—	—	—	—
*Decapoda*	—	Cambaridae	Cambaridae	Cambaridae	Cambaridae	Cambaridae Palaemonidae	Palaemonidae
*Isopoda*	—	Asellota (Suborder)	—	—	—	—	—
*Mysida*	*Hemimysis* (Genus)	*Hemimysis*	*Hemimysis*	*Hemimysis*	*Hemimysis*	*Hemimysis*	*Hemimysis*

**Table-4:** Monthly abundance (numbers) of aquatic insect orders (suborders) in the Shadegan and Hawr Al Azim wetlands, October 2011-September 2012.

Order (Suborder)	2011			2012				Total
	
	Autumn		Winter	Spring		Summer		
			
	October	December	March	April	June	July	September	
*Coleoptera*	25	290	452	705	1153	1888	948	5461
*Diptera*	2	1244	1364	1309	152	123	44	4238
*Ephemeroptera*	7	46	116	15	46	29	31	290
*Hemiptera*	24	28	250	620	509	118	21	1570
*Lepidoptera*	1	4	0	4	1	1	0	11
*Megaloptera*	0	0	1	0	0	0	0	1
*Neuroptera*	1	0	0	0	0	0	0	1
*Odonata (Anisoptera)*	20	23	84	120	89	246	127	709
*Odonata (Zygoptera)*	144	453	1556	1564	1051	428	213	5409
*Plecoptera*	3	0	0	0	0	0	0	3
*Decapoda*	0	86	40	15	1	124	90	356
*Isopoda*	0	29	0	0	0	0	0	29
*Mysida*	5	32	62	47	229	45	36	456
Total	232	2235	3925	4399	3231	3002	1510	18534

**Figure-2:**
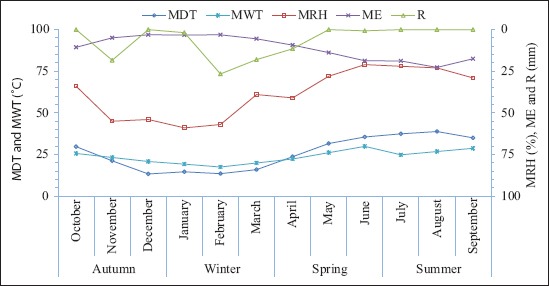
Meteorological data, October 2011-September 2012. MDT=Mean daily temperature, MWT=Mean water temperature, MRH=Mean relative humidity, ME=Mean evaporation, and R=Rainfall.

**Figure-3:**
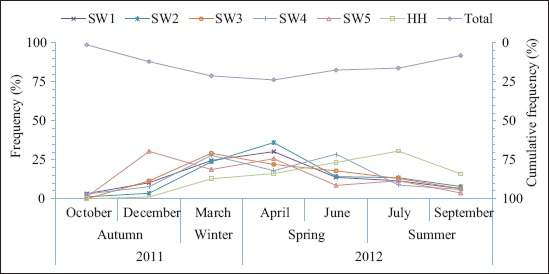
Monthly abundance of the aquatic insects in the Shadegan and Hawr Al Azim wetlands, October 2011-September 2012. SW_1_ to SW_5_=Selected sites of the Shadegan wetland and HH=Hawr Al Azim wetland.

**Figure-4:**
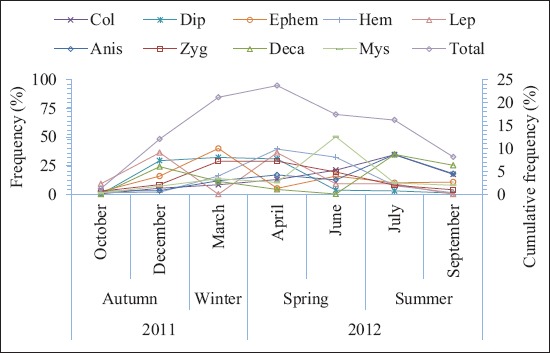
Monthly abundance of aquatic insect orders (suborders) in the Shadegan and Hawr Al Azim wetlands, October 2011-September 2012. Col=*Coleoptera*, Dip=*Diptera*, Ephem=*Ephemeroptera*, Hem=*Hemiptera*, Lep=*Lepidoptera*, Anis=*Odonata*: *Anisoptera*, Zyg=*Odonata*: *Zygoptera*, Deca=*Decapoda* and Mys=*Mysida*.

Figure-2 shows the meteorological data including mean daily temperature, mean relative humidity, and rainfall which were obtained from the Shadegan synoptic weather station. Furthermore, mean water temperature was determined by measuring water temperatures at each sample site of the wetlands. Figures-3 and 4 show the monthly abundance of selected sites and abundance of orders or suborders of aquatic insects in the Shadegan and Hawr Al Azim wetland, respectively. Based on presented results in Tables-2-4 and Figures-2-4, the abundance of aquatic insects gradually increases in association with a reduction of the average daily temperature, increases in the mean relative humidity and precipitation, and reduces the average evaporation from October to April. Conversely, insect abundance gradually decreases in association with increasing average daily temperature, reduction of the mean relative humidity and precipitation, and an increase in the average evaporation from April to September.

The greatest abundance of aquatic insects was observed in April and then in March and June, respectively (Tables-2-4 and Figures-2-4).

**Figure-5:**
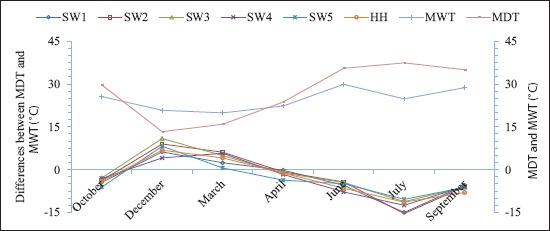
Differences between mean water and daily temperature of the Shadegan and Hawr Al Azim wetlands, October 2011-September 2012. SW_1_ to SW_5_=Selected sites of the Shadegan wetland and HH=Hawr Al Azim wetland, MDT=Mean daily temperature and MWT=Mean water temperature.

Based on differences between mean water and daily temperature presented in Figure-5, the differences between the average daily and water temperatures were a minimum (0.4°C) in April while it was higher in the other months. The lowest abundance of the aquatic insects was observed in October and then in September and December, respectively (Tables-2-4 and Figures-2-4). There were also significant differences by Wilcoxon signed-ranks test between the monthly abundance of the aquatic insects of October with December, March, April, June, July, and September; December with March and April; and September with June and July in the Shadegan and Hawr Al Azim wetlands (p<0.05) (Table-1).

## Discussion

Climate change may affect in various ways. Climate change seems to play a key role in the dissemination of disease [29]. It may expand the geographical distribution and change epidemiology of vector-borne diseases [30-32]. Global climate change may spread and increase the potential risk of vector-borne diseases to temperate regions [33]. Climate change may also affect the host-seeking vector activity periods and lead to increase or decrease vector abundance and distribution [34]. The results of the present study show that the abundance of aquatic insects gradually increases in association with the seasonal decline in average daily temperature, increases in the mean relative humidity and precipitation, and declines the average evaporation from October to April. A large density of *Aedes vittatus* was detected after the onset of the rainy season following the distinct dry season [20]. The environment like climate is a major regulatory agent that may cause to more adaptation with habitat or coevaluation of fungal or bacterial contaminated or increasing habitat infestation and insecticide resistance of cockroaches[35-56].

In the spring, and especially in April, when the difference between the average daily air and water temperatures reaches minimum (0.4°C) (Figure-5), the aquatic insect abundance reaches a maximum (Tables-2-4 and Figures-2-4). The ecological and environmental conditions which are optimum for aquatic insect growth [21,28] also occur at this time. In the spring season, insects mostly change to families that have high-level biological indices (family biotic index; biological monitoring working party; average score per taxon; *Ephemeroptera*, *Plecoptera*, and *Trichoptera* index; and percent contribution of dominant family) due to improving environmental conditions [21]. Furthermore, spring populations of aquatic insects shift to families that are a symbol of clean water such as *Ephemeroptera*, *Plecoptera*, *Trichoptera*, and *Odonata* orders, but in the autumn, usually these families are not seen or their densities are extremely diminished (Table-4 and Figure-4).

Conversely, species abundance gradually decreases in association with an increase of the seasonal average daily temperature and decline of the mean relative humidity and precipitation and increases the average evaporation from April to September (Tables-2-4 and Figures-2-4). In the late summer with the warming weather, increasing evaporation (Figure-2) and water salinity, the declining water quality, and sedimentation of atmospheric dust on the floating aquatic plants and algae, (forming a hard layer on them [21,28]), the habitat becomes less suitable for aquatic insect growth. Consequently, insect abundance decreases and reaches a minimum in the autumn (Figures-2-4). Furthermore, autumn remains a relatively neglected season in wetland ecosystem research. In spite of the importance of autumn events including leaf senescence, fruit ripening, bird and insect migration, and induction of hibernation and diapause, these facts really occur. Altering the reproductive capacity of individuals, exacerbating invasions, allowing pathogen amplification and higher disease transmission rates, reshuffling natural enemy-prey dynamics, shifting the ecological dynamics among interacting species, and affecting the net productivity of ecosystems are also made by changes in autumn phenology [57].

In the fall, populations of aquatic insects also turn mainly to families that they have low-level biological indices, as reflected by the family biotic index; biological monitoring working party; average score per taxon; *Ephemeroptera*, *Plecoptera*, and *Trichoptera*; and percent contribution of dominant family tolerating poor conditions. In fact, in the fall, populations of insects turn to families that are a symbol of unclean water (Table-3). Therefore, we can see that the population changes of aquatic insects are related to the effects of seasonal climatic conditions throughout the year.

The ecological consequences of currently acting ecosystems of stressors arising from human activities are likely to be modified by climate change. In the face of anthropogenic climate change, the problem becomes even more challenging [58]. The trends of increase and decrease year by year of vector-borne diseases support the theory of the role of climate changes [29]. With the on-going climate change, the rainforests are expected to experience severe drought events in the future [59]. Warming climates are facilitating the range expansion of many taxa to habitats of higher latitudes and elevations [60]. Interacting species can respond differently to cause unexpected consequences [61]. An increase in both temperature average and variation had a more intense effect than an increase in temperature average alone [62].

The present study shows an optimum condition for growth of the aquatic insects in the spring. When differences between the average daily and water temperatures were minimal, in April, the aquatic insect abundance reached a maximum and it can be concluded that the population dynamics of aquatic insects were due to the effect of climate seasonality through the year. Climate change is known to have strongly impacted current patterns of genetic variation of animals and plants in Europe. However, ecological factors also have the potential to influence demographic history and thus patterns of genetic variation [63].

## Conclusion

As wetlands are suitable ecosystems for assessing the effect of seasonal climate on the density of aquatic insects, the present study was carried out to consider the effects of such seasonality on populations of aquatic insects in the Shadegan and Hawr Al Azim wetlands between October 2011 and September 2012. The present study showed an optimum condition for the growth of the aquatic insects in the spring (Tables-2-4 and Figures-2-4) when differences between the average daily and water temperatures reach minimum (Figure-5).

## Authors’ Contributions

HN and AS contributed equally to designing, performing, and writing the work. Both authors read and approved the final manuscript.
